# Repetitive Transcranial Magnetic Stimulation With and Without Text4Support for the Treatment of Resistant Depression: Protocol for a Patient-Centered Multicenter Randomized Controlled Pilot Trial

**DOI:** 10.2196/46830

**Published:** 2023-12-07

**Authors:** Medard Kofi Adu, Raquel da Luz Dias, Belinda Agyapong, Ejemai Eboreime, Adegboyega O Sapara, Mobolaji A Lawal, Corina Chew, Karen Diamond Frost, Daniel Li, Michael Flynn, Sameh Hassan, Ahmed Saleh, Sanjana Sridharan, Matt White, Vincent IO Agyapong

**Affiliations:** 1 Department of Psychiatry Dalhousie University Halifax, NS Canada; 2 Department of Psychiatry University of Alberta Edmonton, AB Canada; 3 Alberta Health Services, Addiction and Mental Health Edmonton, AB Canada; 4 Nova Scotia Health Authority Halifax, NS Canada

**Keywords:** repetitive transcranial magnetic stimulation, treatment-resistant depression, cognitive behavioral therapy, Text4Support, text messaging, major depressive disorder

## Abstract

**Background:**

Treatment-resistant depression (TRD) is the inability of a patient with major depressive disorder (MDD) to accomplish or achieve remission after an adequate trial of antidepressant treatments. Several combinations and augmentation treatment strategies for TRD exist, including the use of repetitive transcranial magnetic stimulation (rTMS), and new therapeutic options are being introduced. Text4Support, a text message–based form of cognitive behavioral therapy that allows patients with MDD to receive daily supportive text messages for correcting or altering negative thought patterns through positive reinforcement, may be a useful augmentation treatment strategy for patients with TRD. It is however currently unknown if adding the Text4Support intervention will enhance the response of patients with TRD to rTMS treatment.

**Objective:**

This study aims to assess the initial comparative clinical effectiveness of rTMS with and without the Text4Support program as an innovative patient-centered intervention for the management of patients diagnosed with TRD.

**Methods:**

This study is a multicenter, prospective, parallel-design, 2-arm, rater-blinded randomized controlled pilot trial. The recruitment process is scheduled to last 12 months. It will involve active treatment for 6 weeks, observation, and a follow-up period of 6 months for participants in the study arms. In total, 200 participants diagnosed with TRD at rTMS care clinics in Edmonton, Alberta, and rTMS clinics in Halifax, Nova Scotia will be randomized to 1 of 2 treatment arms (rTMS sessions alone or rTMS sessions plus Text4Support intervention). Participants in each group will be made to complete evaluation measures at baseline, and 1, 3, and 6 months. The primary outcome measure will be the mean change in the scores of the Patient Health Questionnaire-9 (PHQ-9). The secondary outcome measures will involve the scores of the 7-item Generalized Anxiety Disorders Scale (GAD-7), Columbia-Suicide Severity Rating Scale (CSSRS), and World Health Organization-Five Well-Being Index (WHO-5). Patient data will be analyzed with descriptive statistics, repeated measures, and correlational analyses. Qualitative data will be analyzed using the thematic analysis framework.

**Results:**

The results of the study are expected to be available 18 months from the start of recruitment. We hypothesize that participants enrolled in the rTMS plus Text4Support intervention treatment arm of the study will achieve superior outcomes compared with the outcomes of participants enrolled in the rTMS alone arm.

**Conclusions:**

The application of the combination of rTMS and Text4Support has not been investigated previously. Therefore, we hope that this study will provide a concrete base of data to evaluate the practical application and efficacy of using the novel combination of these 2 treatment modalities.

**International Registered Report Identifier (IRRID):**

PRR1-10.2196/46830

## Introduction

### Background

Major depressive disorder (MDD) is one of the most prevailing mental health conditions, which affects about 300 million people globally [1]. MDD is considered the second leading cause of years lived with disability, and it was associated with an estimated worldwide loss of over 63 million healthy years in 2010 [2,3]. The lifetime prevalence of MDD stands at 15% [4], with a substantial socioeconomic impact and an estimated annual cost of about US $236 billion and €91 billion (US $96 billion) in the United States and Europe, respectively [5,6]. The lifetime prevalence of MDD within the Canadian population stands at 9.9% [7], with an estimated annual economic cost of about US $12 billion [8]. Data demonstrate that among MDD patients who receive first-line treatment, about 50% fail to achieve remission, with two-thirds requiring further treatment trials before they can reach the state of remission [9,10].

Most MDD patients may respond to treatment modalities, which include conventional psychopharmacology, psychotherapy, and electroconvulsive therapy. However, about one-third of patients fail to respond to these treatment modalities and hence are diagnosed with treatment-resistant depression (TRD) [11,12]. Owing to the heterogeneity of MDD and the absence of clear biological predictors of what constitutes a response to a specific treatment agent, there is a high chance of any patient with treatment failure being classified as treatment resistant [13,14].

The concept of TRD spans over decades [15]. While many definitions of TRD have been proposed in the literature, the consensus appears to center on the failure of an MDD patient to respond to 2 adequate trials of recommended antidepressant pharmacotherapy [16]. However, the term “adequate response” may be in contention regarding the precise meaning of what constitutes “adequate.” Generally, it has been argued that “TRD” may not be the best term to describe the nonresponse to therapeutic modalities in patients with MDD [16]. Instead, the term “difficult-to-treat depression” has been proposed in the literature. It has the added benefit of preventing MDD patients from feeling that their condition is impossible to treat with the recommended treatment modalities [17]. In Canada, TRD is diagnosed based on nonresponse to at least two antidepressant medications from different classes [18].

Data in the literature suggest that TRD is characterized by a disproportional socioeconomic burden for affected individuals, their relatives, and the health care system in general [19]. TRD presents with a higher direct and indirect medical cost than MDD, which is responsive to therapeutic interventions. This medical cost increases with the severity of TRD [20-23]. Compared with other depressive conditions, TRD leads to at least about 12% more outpatient attendance, is associated with about twice the chances of hospitalization, and is associated with the use of up to about 3 times more psychopharmacological medications [22]. Evidence in the literature suggests that the overall medical cost for hospitalization among patients with TRD is about 6 times greater compared to that among patients without TRD [22]. Similarly, the total cost of a TRD episode among patients with TRD is about 2.7 to 5.8 times higher compared to that among patients without TRD. This difference in cost is due to a longer duration of TRD episodes coupled with a greater rate of usage and a higher cost per treatment visit among patients with TRD [23]. Furthermore, the overall depression-related cost among patients with TRD is estimated to be about 19 times greater compared to that among patients with other depressive conditions [22]. Data suggest that up to about 47% of depression-related costs are attributed to TRD in the United States alone [21,24].

TRD is associated with several risk factors, which include physical or psychiatric conditions, such as anxiety disorders, personality disorders, and hypothyroidism; drug interactions; sociodemographic risk factors, such as individual education attainment and gender; and other external issues affecting ill health [21,25-27]. Furthermore, bipolarity is considered a major factor in TRD based on the inadequate management of bipolar diathesis in MDD, which is diagnosed as unipolar [26,28,29]. Again, TRD is characterized by a greater risk of comorbidities of both psychiatric and medical conditions, an increased risk of suicides, and impairment in normal functioning such as impaired quality of life and social functioning [20,30-32]. Due to its recurrent nature, an estimated 80% of patients with TRD relapse within 1 year after remission of their symptoms [30].

The prevalence of TRD varies significantly across the literature (12%-55%) [21,33-35]. In the United States alone, the estimated annual prevalence of medication-treated MDD was 8.9 million adults, of which 2.8 million (30.9%) had TRD [24]. A study that evaluated the prevalence and burden of TRD among patients with MDD in 4 Latin American countries (Argentina, Mexico, Colombia, and Brazil) revealed that 29% of the study participants with MDD were resistant to medication. The results demonstrated a TRD prevalence of 33% in Argentina, 21% in Mexico, 32% in Colombia, and 40% in Brazil [19]. In Europe, data from a multicenter study of patients with TRD suggested a prevalence rate of 41% among patients with MDD, while in the United Kingdom, a study conducted on patients treated for MDD in a primary care setting demonstrated that 55% of the patients had TRD [36,37]. In Canada, the prevalence of TRD is about 21.7%. However, there were no differences in prevalence between men and women or among ethnic groups [18]. The wide variation in prevalence estimates is not unconnected with the variation in the definition and criteria for classifying TRD in the literature.

### Treatment Options for TRD

Since the idea of TRD was established in 1974 [38,39], there has not been any straightforward standardized technique made available for the management of TRD; however, several studies have been conducted to evaluate the optimum treatment modalities for the same [40,41]. Many different psychopharmacological and nonpharmacological agents have been proposed and considered for the management of TRD. These treatment strategies may consist of psychopharmacological options, such as switching, augmentation, combination, and optimization of antidepressant medications [42]. Other comprehensive strategies include electroconvulsive therapy [43], psychotherapy [44], exercise [45], deep brain stimulation [43], light-based therapy [45], yoga [45], and transcranial magnetic stimulation (TMS) [43].

### Use of Repetitive TMS in the Treatment of TRD

TMS was developed in the late 1980s [46]. Subsequently, researchers came up with newer techniques to deliver multiple pulses of TMS in shorter intervals referred to as repetitive TMS (rTMS) [47]. Thereafter, several studies were conducted to evaluate the efficacy of rTMS for the management of MDD [48] and other psychiatric conditions. rTMS is effective in the management of TRD, with response rates ranging from 30.6% to 64.7% [49-54]. In 2002, the application of rTMS for the management of TRD was approved by Health Canada [16].

rTMS is a noninvasive brain stimulation treatment modality that is deemed to be a valuable and promising treatment technique for TRD [55]. Two main protocols of rTMS are mostly used in the management of TRD: low-frequency (≤1 Hz) rTMS targeting the right dorsolateral prefrontal cortex and high-frequency (5-20 Hz) rTMS targeting the left dorsolateral prefrontal cortex [56]. Studies have argued that there is no significant difference in the efficacy of low-frequency rTMS and high-frequency rTMS in the management of patients with MDD. However, high-frequency rTMS seems better suited in clinical practice owing to the placebo effect [57,58]. The rTMS protocol of choice commonly used in suicide interventions (a common feature of TRD) is high-frequency rTMS [59,60]. The evidence in the literature in support of rTMS as a suicide reduction treatment intervention is modest and yet promising. Although there have been variations in the rTMS protocol among studies, several studies have demonstrated reductions in the rates of suicidality, with remission rates ranging from 40% to 65% in patients diagnosed with depressive disorders [59-63].

A standard session of rTMS for the management of TRD may consist of 20 to 30 daily treatment sessions for 4 to 6 weeks [64]. The administration of rTMS does not require an anesthetic agent and hospitalization, and thus, it is more accessible compared to electroconvulsive therapy in the management of TRD. Furthermore, rTMS application is deemed safe and tolerable, with no serious adverse effects among users [65].

### Digital Technology Used in the Treatment of TRD

Though many treatment interventions for depressive conditions have been proposed in the literature, it is quite obvious that the traditional face-to-face means of delivering mental health care alone will not be enough to meet the demands for health care services, given the high prevalence of MDD and TRD, without any hope or likelihood of a decrease any time soon [66]. According to the 2012 Canadian Community Health Survey on Mental Health, about 2.3 million Canadians mentioned among other things that the barriers hindering them from accessing quality mental health care include a lack of a readily available care system, stigma, and high cost of health care services [67,68]. There has been a significant advancement in the development of digital technologies for the provision of health care in recent decades [69]. In particular, mobile digital information and communication technologies continue to advance at an exponential rate, making it possible to communicate, obtain information, and access care services with ease.

There is evidence in the literature in support of the therapeutic efficacy and cost-effectiveness of these digital technologies, such as tele-mental and e-mental health services, with the potential of enhancing easy access to health care and thereby helping to reduce the decades-old problem of unmet treatment needs and the wide treatment gap for mental conditions, particularly TRD [70,71]. These useful technological methods of health care delivery include smartphone apps, text messages, and email messaging [71,72]. In 2019 alone, about 97% of the world’s population lived in communities with coverage of mobile cellular signals [73]. This exponential growth of mobile networks and phones has greatly opened new opportunities for health care delivery and health promotion, with a significant improvement in mobile health within recent years [74]. A global survey conducted in 2015 demonstrated that Canada, among Western countries, is in the high internet use group, with about 90% of Canadians using internet services and 67% owning a smartphone [75]. In 2022, internet usage among individuals aged 15 years or above in Canada increased to 95% from 92% in 2020. Notably, there was a significant rise in usage among those aged 75 years or older, with an increase from 62% in 2020 to 72% in 2022 [76].

Technology-enabled cognitive behavioral therapy–based programs have been increasingly acknowledged as innovative, accessible, cost-effective, and acceptable ways of delivering psychological treatments to patients and the general public with mental health problems [77]. An estimated 99% of all messages received on mobile phones are opened, and about 90% of all text messages end up being read within 1 to 3 minutes of reception [78]. Thus, providing an affordable means of accessing care could help close the psychological treatment gap for patients with TRD [79].

Text4Support is one of the supportive text messaging programs in the ResilienceNHope [80] suite delivered by the Global Psychological eHealth Foundation [81]. The program allows users to receive daily supportive text messages, which have been written by a team of cognitive behavioral therapists and mental health professionals in collaboration with users of mental health services. The aim of this Text4Support depression program is to positively enhance the mood of patients with clinical depression. The program operates using positive reinforcement to correct distorted or negative thought patterns.

Evidence in support of the therapeutic efficacy of supportive text messaging exists from randomized controlled trials (RCTs) on supportive text messaging for the management of MDD conducted in Canada [82] and Ireland [83]. In these trials, patients with MDD were offered twice daily supportive text messages in addition to their usual care. Overall, there were significantly greater reductions in the symptoms of MDD among patients in the intervention group than among those in the control group receiving only usual care. After 3 months, the study in Canada demonstrated a statistically significant difference in the mean Beck Depression Inventory-II (BDI-II) score between the intervention group and the control group (mean change of −7.6, 95% CI −13.2 to −1.9; Cohen *d*=0.67) in favor of the intervention group [82]. Similarly, the RCT conducted in Ireland demonstrated a statistically significant difference in the BDI-II score between the intervention group and the treatment as usual group (mean change of −7.9, 95% CI −13.06 to −2.76; Cohen *d*=0.85) in favor of the intervention group [83]. User satisfaction with the Text4Support intervention and related programs has been assessed and reported in several other studies [79,84]. About 83% of users of the Text4Mood program reported an overall improvement in their mental health conditions, which they attributed to receiving daily supportive messages [79].

This study seeks to investigate the combined effect of an rTMS intervention with and without a text message–based cognitive behavioral therapy program (Text4Support) in patients diagnosed with TRD. The study also aims to generate effect size data for these interventions, which will help inform sample size and power calculations for a full-scale randomized clinical trial.

## Methods

### Study Design and Setting

**Figure 1:**
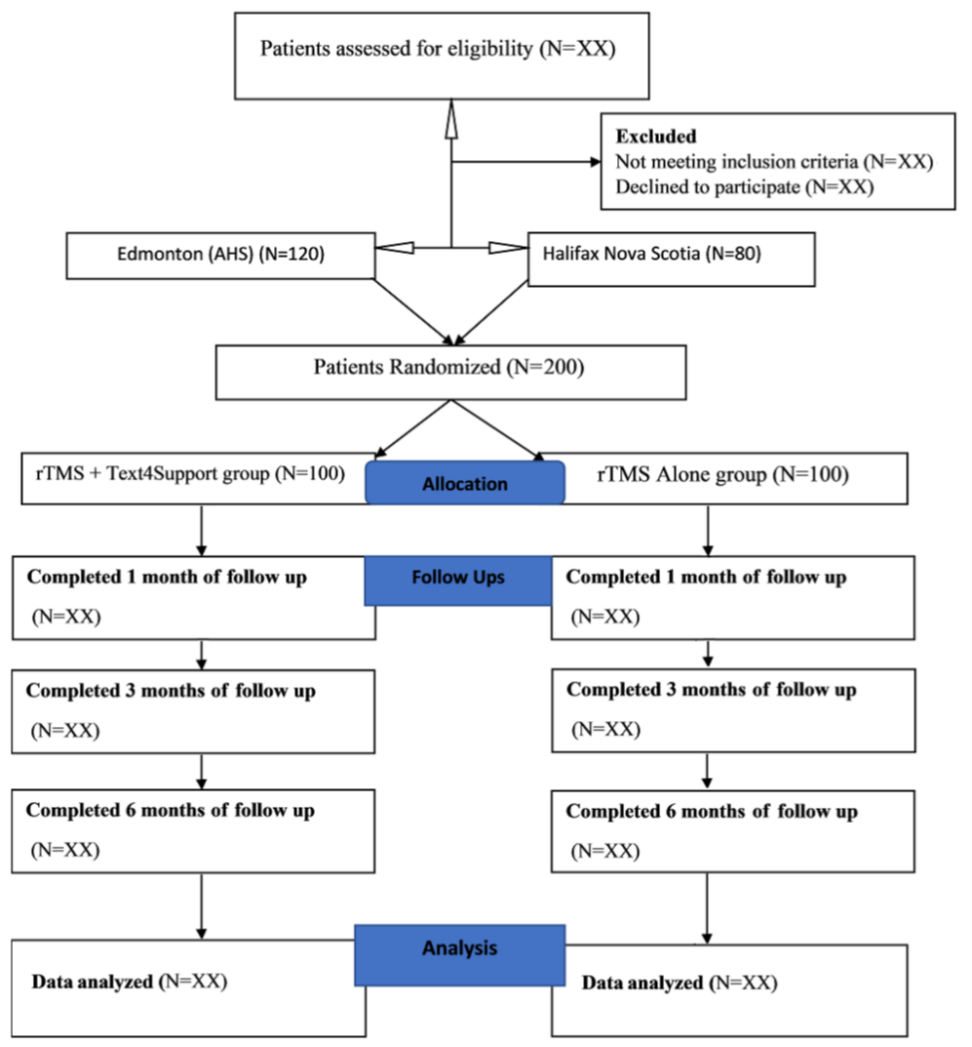
CONSORT (Consolidated Standards of Reporting Trials) flowchart. AHS: Alberta Health Services; rTMS: repetitive transcranial magnetic stimulation.

This study is a multicenter, prospective, parallel design, 2-arm, rater-blinded randomized controlled pilot trial. Participants will be randomly assigned to 1 of 2 treatment conditions. The first condition includes rTMS sessions combined with Text4Support. The second condition includes treatment as usual (rTMS sessions alone). The recruitment process is scheduled to last 12 months. It will involve active treatment for 6 weeks and follow-up with observation periods of 6 weeks, and 3 and 6 months for participants in both arms of the study. Figure 1 presents the CONSORT (Consolidated Standards of Reporting Trials) flowchart for the project. Participants will be recruited from 4 different centers for this project. Two centers (the Addiction and Mental Health Clinic and the Alberta Day Hospital) are from the large sociodemographically diverse city of Edmonton in Alberta, Western Canada. The remaining 2 centers are in Halifax and Annapolis Valley in Nova Scotia, Canada. Table 1 presents the proposed timelines for this project.

**Table 1:** Gantt chart displaying project timelines.

Milestone		Year 1					Year 2	
		Q1	Q2	Q3	Q4	Q1		Q2
**Milestone 1: Recruitment and training of trainees in psychiatry, and setting up of infrastructure for the delivery of Text4Support**								
	1.1. Advertising and recruitment of a trainee in psychiatry who will support the research/evaluation of the project component, apply rTMS^a^, and facilitate the delivery of Text4Support	✓						
**Milestone 2: Recruitment of study participants**								
	2.1. Recruitment, baseline assessment, and randomization		✓	✓				
	2.2. Assignment into 1 of the 2 arms of the study		✓	✓				
	2.3. Delivery of Text4Support and rTMS to participants		✓	✓	✓			
**Milestone 3: Follow-up assessment of study participants**								
	3.1. Follow-up assessment of individual study participants				✓	✓		
	3.2. Follow-up satisfaction survey of participants in all groups				✓	✓		
**Milestone 4: Data compilation, data analysis, and preparation of reports, publications, and presentations**								
	4.1. Data compilation		✓	✓	✓	✓		✓
	4.2. Data analysis		✓	✓	✓	✓		✓
	4.3. Preparation of reports, publications, and presentations							✓

^a^rTMS: repetitive transcranial magnetic stimulation.

### Participants

We aim to randomize 200 patients (Edmonton/AB=120 and Halifax/NS=80) in this study.

#### Inclusion Criteria

The inclusion criteria are as follows: (1) Age 18 years or above; (2) Diagnosis of MDD based on the Diagnostic and Statistical Manual of Mental Disorders, Fifth Edition criteria, and failure of at least two standard antidepressant treatments during the current episode; (3) Good understanding of the English language; (4) Possession of a smartphone, and ability to receive and read text messages; and (5) Ability to consent, and willingness to provide written informed consent.

#### Exclusion Criteria

The exclusion criteria are as follows: (1) Age less than 18 years; (2) Diagnosis (current unless otherwise stated) of a neurological disorder (including a history of seizures), a primary or secondary tumor in the central nervous system, cerebrovascular disease, stroke, cerebral aneurysm, or movement disorder; (3) Diagnosis of a current psychotic disorder, such as substance-induced psychosis, psychotic disorder due to a medical condition, or MDD with psychotic features at the time of screening; (4) Diagnosis of a current personality disorder that may hinder participation in this research or may have the potential of affecting cognition and the ability to fully participate in the study; (5) Presence of a learning disability as identified through medical history or by the investigator during the assessment process; (6) Involvement in any drug or device clinical trial within the last 6 weeks before the screening visit or involvement in another clinical trial for the duration of this study; and (7) Identification of or the sudden appearance of any condition identified by the investigators to have the potential to hinder the progress and completion of the trial, or be a confounding factor for the outcome assessment.

### Sample Size Calculation

This study is a pilot study with no standardized data documented on the effect size, which could aid in power and sample size calculations. We will therefore use data that can be generated from the study participants recruited within the existing protocol. This approach is accepted for pilot studies that consist of novel interventions, such as this, and this method has been described by Haynes et al [85] as using “the participants I can get.” In this regard, the study will be limited to a sample size of about 200 (120 from Edmonton and 80 from Halifax), with 100 participants recruited in each of the study arms. Since TRD is characterized by periods of relapses and depressive attacks, we anticipate a certain number of eligible patients to decline to enroll in the study or even drop out before the completion of the study.

### Recruitment Process

Participant eligibility will be determined by the rTMS specialist team within the various centers. If found eligible for inclusion in the rTMS program, the patient will be approached by a member of the study team to introduce the study. A copy of the information leaflet will be provided to patients to have a detailed insight into the study processes. Once the patients agree to take part in the study, they will be asked to sign a consent form. The signing of the consent form and recruitment process will be conducted face-to-face at the various study sites during the week of the rTMS eligibility assessment of the participants, which usually occurs 1 week before the rTMS sessions begin. Participation in the study is by choice, and a participant can decide to withdraw at any time without providing a reason. To withdraw from the study, participants can contact the research coordinator to communicate their decision to discontinue their participation in the study. Once withdrawn, the study will not collect any new information about them, and if deemed fit, the said participants may approach the research team to withdraw all data collected on them before the study data are analyzed or disseminated.

### Randomization and Blinding

A simple randomization technique will be employed using a single sequence of random assignments. The simple randomization will be performed using a computer-generated Excel sheet (Microsoft Corp). To ensure balance (1:1), the randomization will be stratified by using permuted blocks. The codes generated will then be transmitted via text message by an independent statistician directly to a blinded researcher’s password-protected phone line with a secure online backup. This process will occur for enrollment as each participant signs the consent form.

Since blinding of participants in this study is impossible, their treatment allocation will be made known to them right after the randomization process. Primary outcome assessors will not be granted access to the database that contains the randomization codes, and they will not be involved with all discussions concerning the study participants and may be blinded to the treatment arm allocations. The data that would be generated from the study will undergo a blind review for the reason of finishing the planned analysis.

### Interventions

Study participants will be randomized to 1 of 2 treatment interventions (rTMS alone or rTMS plus Text4Support). During the introductory visit to the study sites, participants in both arms of the study will be given a detailed orientation about the rTMS application, and all procedures and activities within each visit will be explained to them. As part of their participation in the rTMS program, all patients will be made to complete some standard measures. Participants will be invited to the various rTMS clinics a week before the commencement of the rTMS sessions to enable them to undergo motor threshold (MT) assessment. MT assessment is essential in that it aids in the selection of the required stimulation intensity for each patient for inclusion in the treatment. It is a measure of the minimum intensity of TMS output needed to elicit a motor response in the participating TRD patients in at least 50% of all attempts.

All MT assessments will be conducted by the rTMS team of experts within the rTMS clinics in the various treatment centers, and the team may involve psychiatrists, nurses, and other health care professionals with the requisite training to perform the assessments. Each TMS assessment session will take about 3 to 5 minutes, and the total time will be within 35 to 45 minutes. This visit timeline will be the same for all study participants. Overall, all study participants will be scheduled to receive 30 sessions of rTMS treatment for 6 weeks according to the schedule pre-established by the Alberta Health Services’ Strategic Clinical Network for Addiction and Mental Health and Nova Scotia Health. The rTMS protocol includes low-frequency (1 Hz) inhibitory stimulation targeting the right dorsolateral prefrontal cortex at 120% MT, with 20 trains (60 pulses/train at 60 s) and a total pulse of 1200 for 20 minutes, and high-frequency (10 Hz) excitatory stimulation targeting the left dorsolateral prefrontal cortex at 120% MT, with 75 trains (intertrain interval of 11 s) and a total pulse of 3000 for 19 minutes. The study will use the MagVenture rTMS machine with a figure-of-8 coil. The angle between the coil and the head will be measured at 45 degrees. The cortex area will be targeted by using the 5-cm technique. The prefrontal cortex stimulation site will be determined as 5 cm anterior further ahead of the motor strip in the parasagittal line. Regarding the issue of adherence to the treatment protocol, the rTMS team of experts will contact each patient to remind them of their sessions whenever they default in order to ensure adherence to the treatment. The patients will be continuously monitored, and an attending nurse will remain with each patient during the whole session, checking on any expected side effects. Complete treatment will be considered as attending at least 25 out of 30 sessions.

Additionally, participants in the rTMS plus Text4Support group of the study will be assisted by a study team member to register into the Text4Support program. The process will require all participants in this arm of the study to input their phone numbers into the Text4Support platform, which will be used to deliver daily messages. Starting a day after enrollment, participants will receive daily supportive text messages designed by mental health therapists, clinical psychologists, psychiatrists, and mental health service users. These messages will be based on cognitive behavior therapy principles crafted to positively enhance the mood of users with depressive symptoms and other related mental health problems of concern. The messages will be preprogrammed into a software program that will deliver the messages automatically to the participants’ mobile phones at 10 AM (Mountain Time) and 12 PM (Atlantic Time), and each participant will receive these messages continuously for 6 weeks.

### Hypothesis

We hypothesize that participants enrolled in the rTMS plus Text4Support treatment arm of the study will achieve superior outcomes compared with the outcomes of participants enrolled in the rTMS treatment alone arm of the study in terms of all outcome measures that will be assessed.

### Data Collection and Follow-Up Assessment

All study participants who provide informed written consent and agree to participate in the study will be asked to complete a baseline assessment questionnaire that captures both demographic and clinical information. Study participants will also be asked to complete various follow-up assessments related to primary and secondary outcome measures at 6 weeks, and 3 and 6 months from enrollment. All self-completed surveys will be completed online on tablets or on participants’ cell phones. Clinician-rated scales will be completed either face-to-face at the study sites or over the phone/video conference with the help of a blinded researcher. All follow-up survey links will be delivered to participants’ cell phones at the designated time points. Two reminder text messages will be sent a day apart to encourage participants to complete the follow-up surveys. Participants who do not complete the online surveys despite receipt of 2 reminder text messages will be contacted by a research team member to ascertain if there are any barriers to survey completion and if any assistance is needed. Qualitative data collection will be in the form of a patient experience questionnaire and a key informant interview via telephone, which will be conducted at 3 and 6 months. Data related to participants’ clinic or program attendance rates and use of health services will be compiled from administrative records by a blinded researcher at 6 months.

### Outcome Measures

The primary outcome measure for the study will be changes in the mean score of the Patient Health Questionnaire-9 (PHQ-9) [86] from baseline to the specified time points for the intervention and control groups. With 9 items, the PHQ-9 is half the length of many other depression measures, has comparable sensitivity and specificity, and is made up of the actual 9 criteria upon which the diagnosis of depressive disorders is based according to the Diagnostic and Statistical Manual of Mental Disorders, Fourth Edition. This instrument has the potential to establish major depressive disorder diagnosis, as well as grade the severity of depressive symptoms [86]. The PHQ-9 has good psychometric qualities with high internal consistency, and Cronbach α values of .86 and .89 were obtained in a study that included 2 separate patient populations [87]. The secondary outcome measure will include the 7-item Generalized Anxiety Disorders Scale (GAD-7) [88]. The GAD-7 score is calculated by assigning scores of 0, 1, 2, and 3 to response categories. The GAD-7 total score for the 7 items ranges from 0 to 21, with a score of 0-4 indicating minimal anxiety, 5-9 indicating mild anxiety, 10-14 indicating moderate anxiety, and 15-21 indicating severe anxiety. The GAD-7 has excellent internal consistency (Cronbach α=.92) and test-retest reliability (intraclass correlation=0.83) [88]. The screen version of the Columbia-Suicide Severity Rating Scale (CSSRS) [89] will be included to measure the suicidal ideation of participants. The CSSRS is made up of 6 questions. Users are required to respond “Yes” or “No” to whether they have thought about suicide, have acted or plan to act, or have attempted or plan to attempt suicide. Each of the 6 questions evaluates a different component of the respondent’s suicidal ideation severity and behavior. This measuring tool is scored as low, moderate, or high risk, depending on positive answers (Yes) to the various questions. If a respondent answers positively (Yes) to question 2, the respondent is instructed to respond to questions 3-5. If a respondent answers “No” to question 2, the respondent may skip to question 6. Responding “Yes” to any of the 6 items may imply a need for referral to a mental health care professional, but responding “Yes” to question 4, 5, or 6 indicates a high risk. The CSSRS intensity of ideation subscale has good internal consistency, with Cronbach α values ranging between .73 and .93 [89]. Additionally, the World Health Organization-Five Well-Being Index (WHO-5) [90] will be employed to measure the quality of life of participants. The WHO-5 is a psychometrically robust questionnaire with high internal consistency (Cronbach α=.923) [91]. The WHO-5 is a short questionnaire made up of 5 simple and noninvasive questions, which assess the subjective well-being of respondents. A respondent is asked to rate how well each of the 5 statements applies to them within the last 14 days. Each of the 5 items is scored from 5 (all of the time) to 0 (none of the time). The raw score therefore theoretically ranges from 0 (absence of well-being) to 25 (maximal well-being). To translate the scores to percentages (from 0 [absent] to 100 [maximal]) as required by scales measuring health-related quality of life, the raw score is multiplied 4. The outcome measures and time points for the study are demonstrated in Table 2.

**Table 2:** Client-oriented outcome measures and time points.

Variable type and construct		Tool	Rater	Time points assessed			
				Baseline	1 month	3 months	6 months
**Symptom variables**							
	Depression	Patient Health Questionnaire (PHQ-9)	Client	✓	✓	✓	✓
	Anxiety	Generalized Anxiety Disorders Scale (GAD-7)	Client	✓	✓	✓	✓
	Suicidal ideation	Columbia-Suicide Severity Rating Scale (CSSRS)	Clinician	✓	✓	✓	✓
**Functional variables**							
	Quality of life	World Health Organization-Five Well-Being Index (WHO-5)	Client	✓	✓	✓	✓

### Data Analysis

SPSS Version 26 (IBM Corp) will be used to analyze all quantitative data in accordance with the CONSORT guidelines [92]. Following an intention-to-treat approach, data will be analyzed with descriptive statistics, repeated measures, and correlational analyses. Descriptive data for baseline parameters will be presented using frequencies and percentages among the 2 intervention groups and compared using the chi-square test for categorical variables and the independent sample *t* test for continuous variables. The type of intervention (rTMS alone and rTMS plus Text4Support) will be considered the independent variable, while the scores of the various scales at each time point (6 weeks, and 3 and 6 months) will be considered the dependent variables for each of the analyses. Baseline scores of the respective scales will be used as covariates in the analyses.

To compare the effectiveness of the treatment interventions for the management of TRD among participants, the 1-way between-group analysis of covariance (ANCOVA) comparing changes in the mean scores from baseline to 6 weeks, and 3 and 6 months for all measures will be employed. Preliminary checks will be conducted to ensure there is no violation of the assumption of normality, linearity, homogeneity of variance, homogeneity of regression slopes, or reliable measurement of the variate before ANCOVA is conducted. For participants with missing data, the last observation will be imputed before performing sensitivity analyses of covariance to explore the impact of the imputation of data loss on the PHQ-9 and the secondary measures at each time point.

The chi-square test or Fisher exact test will be employed to compare the prevalence of depression symptoms using the PHQ-9, the prevalence of anxiety symptoms using the GAD-7, suicidal ideations using the CSSRS, and quality of life using the WHO-5 between the 2 intervention groups at all time points in the overall sample.

Furthermore, a paired *t* test will be used to examine the changes in the mean scores of the PHQ-9, GAD-7, CSSRS, and WHO-5 at baseline and 6 weeks for participants who complete the instruments at both time points. Frequencies and percentages will be used to report categorical variables, while mean scores, confidence intervals, and effect sizes will be used to report continuous variables. The 2-tailed α-level criterion for statistical significance will be set at *P*≤.05.

Qualitative data obtained through audio recordings from participant focus group discussion sessions will be transcribed and analyzed using thematic analysis aided by NVIVO software [93]. Data analysis will be both deductive and inductive. After familiarization with the transcripts obtained, 2 researchers will independently conduct the initial coding, and subsequently, the final coding criteria will be agreed by the 2 researchers. This will be followed by an iterative review of emerging themes as informed by the research questions. The final sets of themes and subthemes in relation to the overall study aims will be reported and supported with verbatim quotes.

### Ethical Considerations

The study will be conducted according to the Declaration of Helsinki (Hong Kong Amendment) [94]. The study participants will be made to provide informed consent before their inclusion in the study. The research team will strictly adhere to the protection of participants’ health information according to the structure laid down by the 2 major health institutions (Alberta Health Services and Nova Scotia Health Authority). An independent monitor will be assigned by the research team to monitor the progress of the research, ensure the completeness of informed consent and data safety, and liaise with the ethics committee. Although not anticipated, serious adverse events will be reported to the Research Ethics Committee. The study has received ethical clearance from the Health Ethics Research Board of the University of Alberta (Pro000122696) and the Nova Scotia Health Research Ethics Board (REB File #: 1028488).

## Results

The study is registered at ClinicalTrials.gov (registration number: NCT05570344). The study is yet to commence recruitment of participants. The study results are envisioned to be available 18 months from the start of recruitment. The results are expected to be disseminated at different levels, including practitioners, health care organizations, and academics/researchers. Before the study ends, the team of investigators will organize an engagement plan to discuss the practicability and effectiveness of the study.

## Discussion

### Overview

The findings from the study will provide the data needed to assess the initial effectiveness of rTMS plus Text4Support for individuals diagnosed with TRD. Though there are several pieces of evidence in the literature in support of the effectiveness of rTMS in TRD, the data generated regarding rTMS application are relevant only when it is used as a single treatment modality. However, the concomitant application of psychotherapy with rTMS is limited. Therefore, we hope that this novel project will provide a concrete base of data to enable the evaluation of the wider-scale practical application and efficacy of the combination of rTMS and Text4Support. To the best of our knowledge, this study will be the first clinical trial to investigate the combined effect of these 2 interventions. Owing to the limited availability of data regarding this specific area of treatment, another objective will be to generate effect size information for these interventions to aid future power and sample size calculations for a full-scale RCT.

### Strengths of This Study

This study has several strengths. First, since this RCT will be conducted in a naturalistic clinical care setting, translation of the results to clinical practice will be easier. Regardless of the outcome of this trial, the findings can inform clinical decision-making and aid in choosing the right treatment for a patient with TRD. Second, this study will have a longer follow-up duration than other rTMS trials. Since data up to 6 months from the start of the study will be available, the durability of the treatment effects of rTMS in the longer term can be evaluated. Third, randomizing participants will ensure that participants in the 2 treatment arms have similar psychiatric morbidity at baseline. Moreover, the blinding of all primary outcome assessors to the primary outcome measures will ensure the elimination of rater bias in the outcome measures.

### Limitations

As eligible patients will ultimately decide on whether they want to join the study, a major limitation may be selection bias, which will potentially result in the selection of highly motivated patients. This may affect the ability to achieve a good study sample size and power. Another limitation may be associated with possible variabilities in illness duration and concomitant treatments being received by patients outside the rTMS treatment, which could have confounding effects on the outcomes of our interventions.

## Abbreviations

ANCOVA: analysis of covariance
BDI-II: Beck Depression Inventory-II
CONSORT: Consolidated Standards of Reporting Trials
CSSRS: Columbia-Suicide Severity Rating Scale
GAD-7: 7-item Generalized Anxiety Disorders Scale
MDD: major depressive disorder
MT: motor threshold
PHQ-9: Patient Health Questionnaire-9
RCT: randomized controlled trial
rTMS: repetitive transcranial magnetic stimulation
TMS: transcranial magnetic stimulation
TRD: treatment-resistant depression
WHO-5: World Health Organization-Five Well-Being Index
